# Duality between Time Series and Networks

**DOI:** 10.1371/journal.pone.0023378

**Published:** 2011-08-11

**Authors:** Andriana S. L. O. Campanharo, M. Irmak Sirer, R. Dean Malmgren, Fernando M. Ramos, Luís A. Nunes. Amaral

**Affiliations:** 1 Laboratory for Computing and Applied Mathematics, Instituto Nacional de Pesquisas Espaciais, São José dos Campos, São Paulo, Brazil; 2 Department of Chemical and Biological Engineering, Northwestern University, Evanston, Illinois, United States of America; 3 Department of Computer Engineering, Pontifícia Universidade Católica de Minas Gerais, Belo Horizonte, Minas Gerais, Brazil; 4 Datascope Analytics, Chicago, Illinois, United States of America; 5 Center for Earth System Science, Instituto Nacional de Pesquisas Espaciais, São José dos Campos, São Paulo, Brazil; 6 Northwestern Institute on Complex Systems, Northwestern University, Evanston, Illinois, United States of America; 7 Howard Hughes Medical Institute, Northwestern University, Evanston, Illinois, United States of America; University of Maribor, Slovenia

## Abstract

Studying the interaction between a system's components and the temporal evolution of the system are two common ways to uncover and characterize its internal workings. Recently, several maps from a time series to a network have been proposed with the intent of using network metrics to characterize time series. Although these maps demonstrate that different time series result in networks with distinct topological properties, it remains unclear how these topological properties relate to the original time series. Here, we propose a map from a time series to a network with an approximate inverse operation, making it possible to use network statistics to characterize time series and time series statistics to characterize networks. As a proof of concept, we generate an ensemble of time series ranging from periodic to random and confirm that application of the proposed map retains much of the information encoded in the original time series (or networks) after application of the map (or its inverse). Our results suggest that network analysis can be used to distinguish different dynamic regimes in time series and, perhaps more importantly, time series analysis can provide a powerful set of tools that augment the traditional network analysis toolkit to quantify networks in new and useful ways.

## Introduction

In the context of dynamical systems, time series analysis is frequently used to identify the underlying nature of a phenomenon of interest from a sequence of observations and to forecast future outcomes. Over time, researchers accumulated a large number of time series analysis techniques, ranging from time-frequency methods, such as Fourier and wavelet transforms [1]–[3], to nonlinear methods, such as phase-space embeddings, Lyapunov exponents, correlation dimensions and entropies [4]–[6]. These techniques allow researchers to summarize the characteristics of a time series into compact metrics, which can then be used to understand the dynamics or predict how the system will evolve with time.

Obviously, these measures do not preserve all of the properties of a time series, so there is considerable research toward developing novel metrics that capture additional information or quantify time series in new ways [7]–[10]. One of the most interesting advances is mapping a time series into a network, based on different concepts such as correlations [11], [12], visibility [13], [14], recurrence analysis [15], transition probabilities [16]–[18] and phase-space reconstructions [19], [20] (a complete list of all the proposed maps can be found in Donner *et al*.,(2010) [21] and references therein). These studies have demonstrated that distinct features of a time series can be mapped onto networks with distinct topological properties. This finding suggests that it may be possible to differentiate properties of time series using network measures. However, it remains unclear, for example, how these topological properties relate to the original time series.

At the root of this issue is the fact that most of these maps 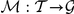 from the time series domain 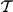 to the network domain 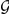 do not have a natural inverse operation 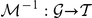. Recently, some attempts to construct an invertible map have been proposed [18], [22], [23]. However, they are either sensitive to arbitrarily chosen parameters [22], [23] or they use information obtained from a given map 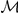 to build an inverse operation 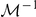 [18]. Consequently, they are not applicable to real world networks, where 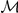 is not known in advance.

A fully invertible map makes it possible to create a “dual” representation of a time series and its network counterpart and directly relate common network statistics back to the original time series and vice-versa. This dual representation would not only allow time series analysis to benefit from the recent surge in network related research [24], [25], but network theory would be able to draw on more than three centuries of theoretical and applied developments in time series analysis. In this paper, we take a significant step toward realizing this goal by introducing a map from time series to networks that has a natural and robust inverse.

## Methods

**Figure 1:**
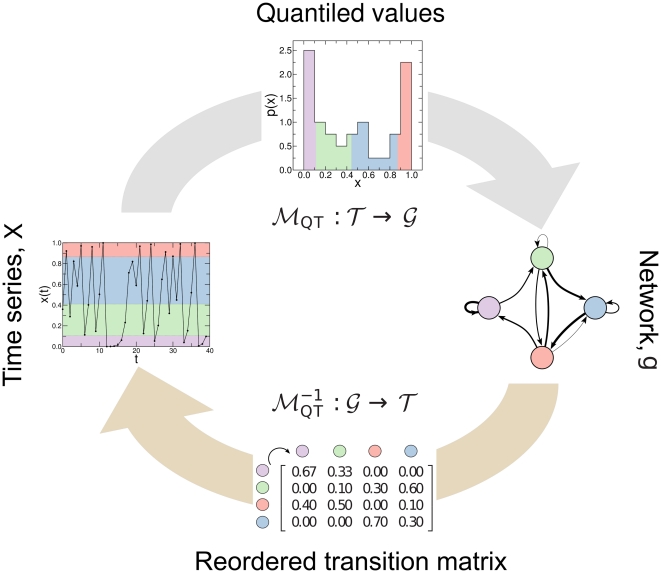
Illustration of the proposed map. *Forward map*: A time series 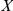 is split into 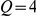 quantiles (colored shading) and each quantile 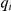 is assigned to a node 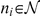 in the corresponding network 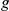. Two nodes 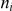 and 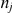 are then connected in the network with a weighted arc 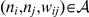 where the weight 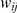 of the arc is given by the probability that a point in quantile 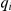 is followed by a point in quantile 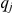. Repeated transitions between quantiles results in arcs in the network with larger weights (represented by thicker lines). *Inverse map*: The weighted adjacency matrix 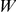 of network 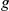 is first normalized such that it is a Markov transition matrix with 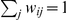. The association between nodes and quantiles is obtained by reordering 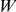 to have large 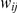 near to the diagonal such that the resulting time series is as continuously smooth as possible [29]. The time series is constructed by repeatedly moving from node 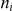 to node 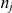 with probability 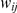 and choosing a random number from the corresponding quantile 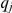 until we have obtained a time series of length 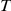.

Let 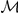 be a map from a continuous time series 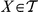 to a network 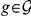, where 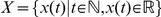 and 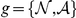 consists of a set of nodes 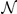 and arcs 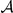. Ideally, such a map would preserve *all* information of the original time series, possibly by a bijective map 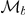 where each time series 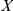 maps to exactly one network 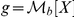 that is invertibly mapped to the exact same time series 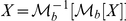. In practice, this is impossible; continuous time series have uncountably many values whereas networks are limited to a countable set of nodes 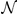 and connections 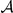 between them. Thus, any map from a continuous time series 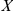 to a network 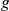 must discretize the time series in some manner. Here, we use a simple discretization of 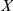 that is not sensitive to the distribution of its values. Specifically, given a time series 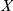, we identify its 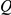 quantiles and assign each quantile 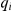 to a node 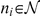 in the corresponding network. Two nodes 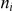 and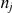 are then connected in the network with a weighted arc 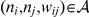 where the weight 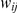 of each arc is the transition probability in a Markov model estimated from the aggregate time series (Fig. 1).

The proposed map, here denoted by 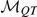, has two important properties. First, it is surjective. Given a time series 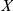 with 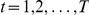 points and the number of quantiles 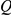, the map will produce one and only one network 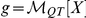. Note that distinct time series 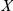 and 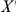 *can* be mapped onto the same network 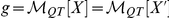 although the network space is large enough that this does not typically happen in practice. Second, if 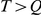, the resulting network is weighted, directed and connected. Third, 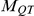 is insensitive to the distribution of values of 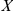. The “forward” map only requires the specification of the parameter 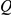. This is in contrast to the maps proposed earlier, where the structures of the resulting networks are very sensitive to the choice of several parameters like time delay, embedding dimension and threshold distance; demanding expert guesses commonly used in techniques like phase-space reconstruction and recurrence analysis [26]–[28].

The map proposed here has the significant advantage that it has a “natural” inverse operation – a realization of a random walk on the network with transition probability 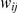 given by the weighted adjacency matrix 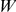 such that 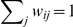 (Fig. 1). Starting from a random node, we construct a time series by performing a random walk in which the probability of moving from node 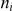 to node 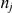 is 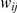. If we identify each node in the network with a particular quantile in the resulting time series 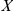, we can construct the time series by dividing its domain into 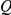 quantiles and for each step of the random walk choosing a value within the corresponding quantile at random with uniform probability. In the absence of *a priori* knowledge of a direct correspondence between quantiles and nodes we assume smoothness in the resulting time series. In this way, nodes can be associated to quantiles by reordering the weighted adjacency matrix 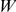 to have large 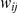 near to the diagonal [29] such that the resulting time series is as “smooth” as possible – a property that is common to many empirical time series. To find the ordering of 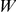 close to the optimal ordering, we use simulated annealing [30] with a cost function that weights each element by its distance to the diagonal [31]:
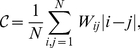
(1)where 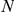 is the order of the transition probability matrix.

For every iteration in the simulated annealing search, we use 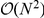 moves in which segments of contiguous nodes attempts to change positions in the ordering. We accept or reject each attempted move following a standard Metropolis algorithm. For each attempt, we randomly pick: (a) a segment of contiguous nodes and (b) a new position for the first node – the remaining nodes will be placed keeping the order relative to the first node. The first node and its new position are picked from a uniform distribution; the width of the segment is picked from a Gaussian distribution whose variance depends linearly on both the temperature 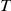 and the size of the network 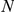 – for low temperatures only changes of single nodes are proposed. We compute the value of the cost function for the new order 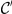 and we accept the change with probability 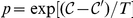 [29].

Like 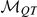, the proposed inverse map, here denoted by 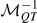, has several important properties. It is also surjective; given a network 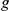 the map will produce a time series 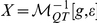 over a realization 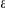, but distinct networks 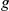 and 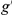 *can* be mapped onto the same time series 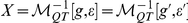. However, it is not strictly one-to-one since it has a stochastic element. That is, 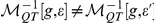. Note that even though the proposed map is not one-to-one, the time series obtained by applying the inverse map with different realizations will have very similar properties. In contrast, previous inverse maps [22], [23] depend on the arbitrary choice of node labels and the resulting time series are highly sensitive to this choice.

## Results

**Figure 2:**
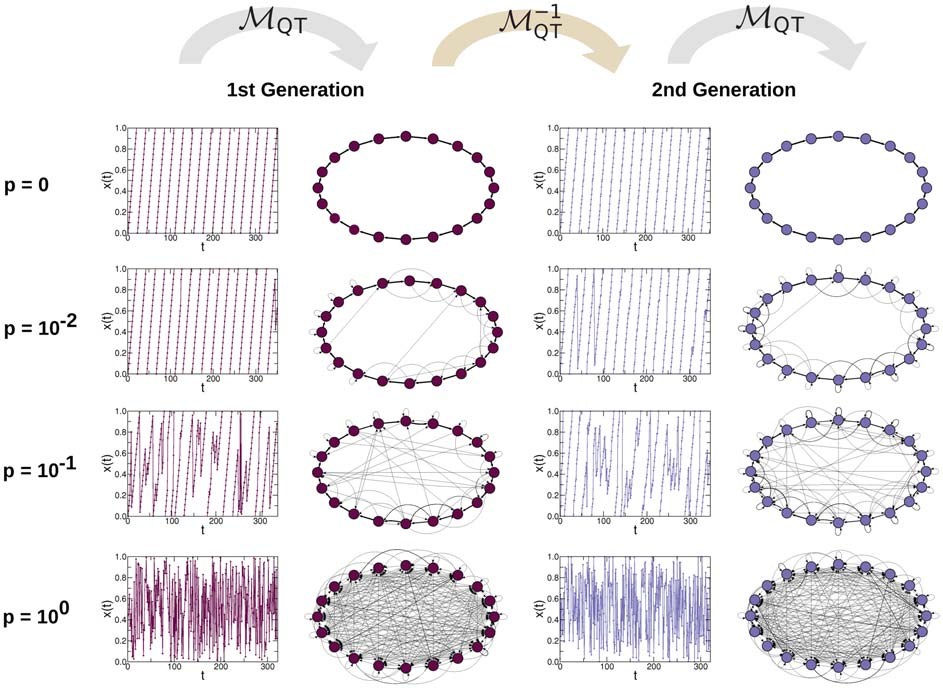
Qualitative assessment of the faithfulness of the proposed map and its inverse. We generate first generation time series from the toy time series model (Eq. 2) ranging from periodic (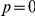) to random (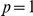) with 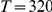 and 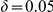. We then construct the first generation networks using 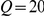 quantiles by applying 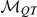 from the corresponding time series. Time series with different values of 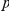 result in networks with different topologies. As the toy time series becomes more random, the corresponding networks also become increasingly random. We construct the second generation time series and the second generation networks by sequentially applying 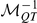 and 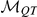, respectively. These panels suggest that the first and second generation time series and networks have similar properties, supporting the hypothesis that it may be possible to use time series analysis to characterize the topology of networks and networks analysis to characterize the structure of time series.

To verify the extent to which the properties of the original time series or network are recovered when 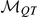 and 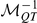 are applied sequentially, we introduce an ensemble of time series that range from periodic to random:
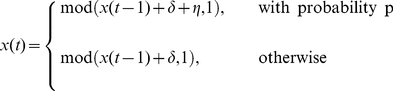
(2)where 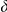 is a constant, 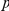 parameterizes the probability that noise modifies the otherwise periodic time series, and 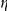 is a random variable drawn from a uniform distribution in 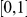. We choose 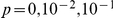 and 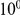 and 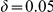 and generate numerous time series with 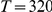 points. We then apply the forward map with 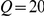 quantiles to the generated time series and obtain the resulting networks. We refer to these time series and networks as the “first generation” time series and networks, respectively. Figure 2 shows that time series with different properties are mapped onto networks with visually distinct topologies. Specifically, as the time series become more random, the corresponding networks become increasingly more random, much like the small-world network model of Watts & Strogatz [32].

**Figure 3:**
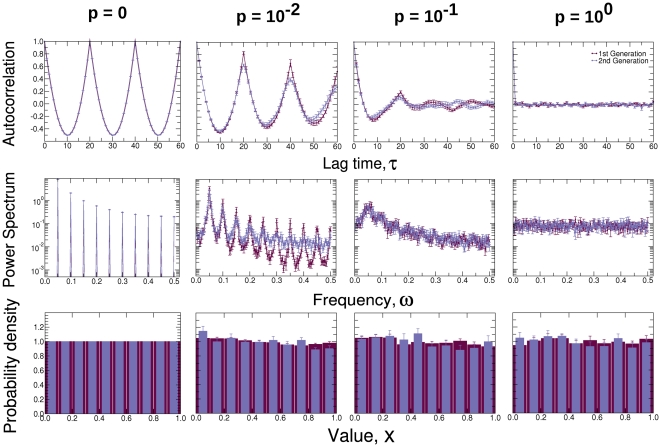
Comparison of statistical properties of first generation and second generation time series. We compare the means of these properties over 10 different realizations of first and second generation time series. Error bars denote standard deviation across realizations. For both the first and second generation time series, the autocorrelation function and the power spectrum reveal a distinct signal when the time series are periodic (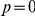), which disappears when the time series become random (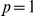). As expected from the toy model that has no biases toward particular values, both the first and second generation time series have values that are uniformly distributed between 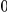 and 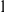 for all values of 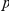.

We next apply the map 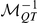 to each of the first generation networks and obtain the “second generation” time series, again with 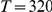 points. For simplicity, we assign each quantile to the corresponding quantile from the first generation time series. The visual similarity between the first generation time series 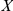 and the second generation time series 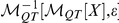 is apparent, regardless of the value of 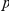 (Fig. 2). We quantitatively demonstrate the faithfulness of the proposed map in the time series domain 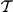 by comparing the autocorrelation function, the power spectrum and the distribution of the first and second generation time series (Fig. 3).

**Figure 4:**
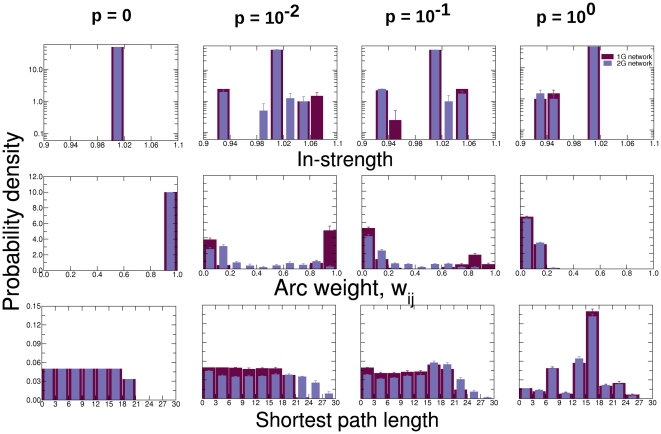
Comparison of topological properties of first generation and second generation networks. We compare the means of these properties over 10 different realizations of first and second generation networks. Error bars denote standard deviation across realizations. In-strength is unitary for every node when the first and second generation networks are regular (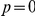) and, as the networks become increasingly random (left to right), the in-strength distribution broadens due to the redistribution of the weights. Note that the out-strength of a node is unitary in all cases, since the weights are Markovian probabilities. Arc weights are unitary for every arc when the first and second generation networks are regular (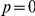). As 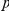 increases, the arc weight distribution of the first and second generation networks shows presence of small weights (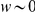) as well as large weights (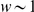). The shortest path length are calculated as the minimum sum of inverted weights on a path from one node to another. Shortest path lengths [25] are uniformly distributed when the first and second generation networks are regular (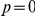). As 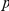 increases, random shortcuts generally decrease the distance between nodes, although for some cases, larger path lengths also arise due to redistribution of weights on the shortest path to other nodes. As the networks become more random (left to right), the shortest path distribution becomes increasingly peaked.

Finally, we apply 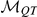 to the second generation time series using 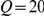 quantiles to obtain the corresponding “second generation” networks. It is visually apparent that first generation networks 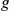 and second generation networks 
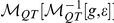
 have similar topologies for all values of 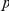 (Fig. 2). We quantitatively demonstrate the faithfulness of the map in the network domain 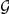 by comparing the in-strength, arc weight and shortest path length distributions of the first and second generation networks (Fig. 4). Our results show that the topological features of the first generation networks are recovered in the second generation networks for all values of 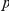. The results of Figures 3 and 4 indicate that our method is able to preserve both structured and unstructured information in both the time series and network domains, even after successive mappings.

**Figure 5:**
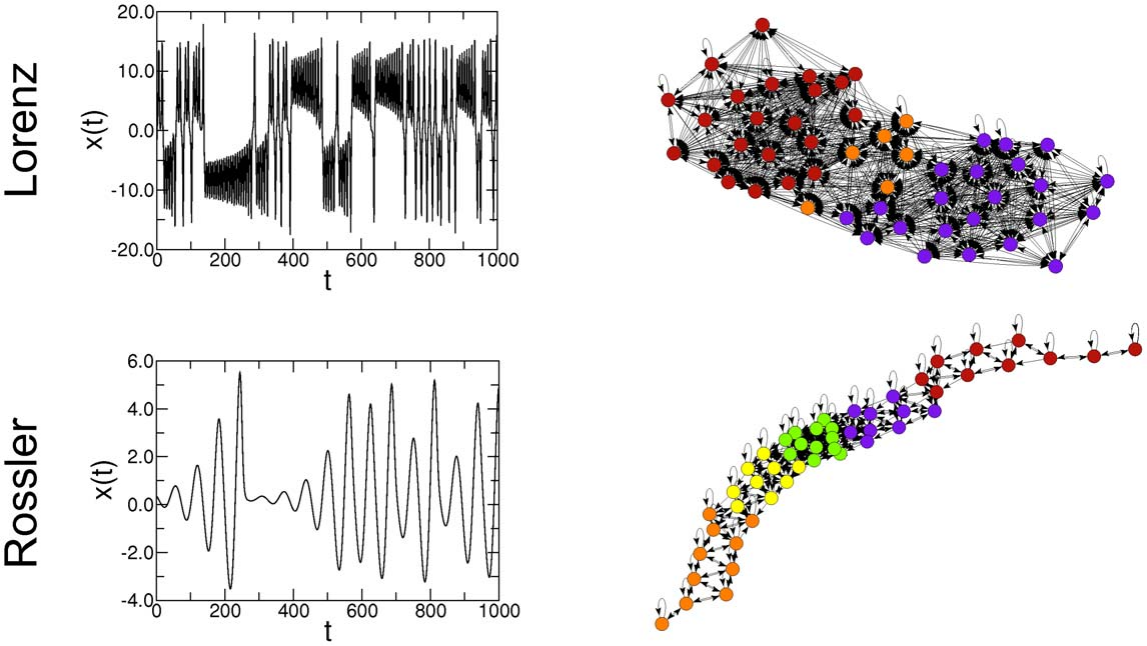
Illustration of the forward map 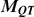 to chaotic time series from Lorenz and Rossler systems. We use 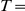
10,000 time points of the 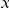 variable of the chaotic Lorenz and Rossler equations and construct networks using 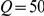 quantiles by applying the forward map. Each node is colored according to the module to which it belongs. The resulting networks display clear differences in topologies. The network of Lorenz's system is bulky with two large modules. It has a modularity value of 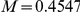, that is much larger than the mean (standard error) modularity value 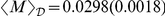 obtained from networks built from the randomizations of the original time series. Furthermore, the two lobes of the Lorenz attractor are mapped into the two largest connected modules in the network. On the other hand, the network of Rossler's system presents an elongated, chain-like pattern due the strong periodicity present in its corresponding time series. The network of Rossler's system is also modular, with five small modules and it has a modularity value of 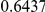. This value is much larger than the mean (standard error) modularity value 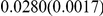 obtained from networks built from the randomizations of the original time series.

To further highlight the potential of the forward map described above, we apply it to two time series belonging to different dynamical systems. The first time series is the 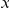 variable of the chaotic Lorenz equations:
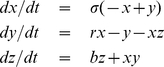
(3)with parameter values 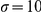, 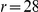, and 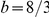. Numerical solutions of these equations leads to an attractor embedded in a three-dimensional space with coordinates 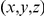 [33]. The trajectory rotates about one of two unstable fixed points and eventually escapes to orbit the other fixed point. This behavior is recognizable in the 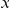 variable (left panel in Fig. 5) since its values oscillate between the positive and the negative 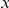-region.

The second time series is the 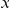 variable of the chaotic Rossler equations:
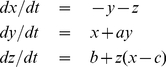
(4)with parameter values 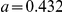, 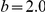, and 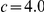. Its phase-space generates a chaotic attractor with a single lobe, in contrast to the Lorenz attractor which has two. The trajectory within the attractor follows an outward spiral close to the 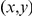 plane around an unstable fixed point. Once the trajectory spirals out enough, a second fixed point influences it, causing a rise and twist in the 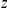-dimension [34]. This behavior generates a quasiperiodic oscillatory pattern in the 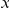 variable, with max/min peaks/troughs with different amplitudes (left panel in Fig. 5).

In both cases, we apply the forward map with 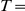
10,000 and 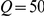 quantiles. The resulting networks (right panel in Fig. 5) display clear differences in topology. The network of Lorenz's system presents a bulky structure, with the two lobes of the Lorenz attractor being mapped into the two largest connected modules in the network. On the other hand, the network of Rossler's system presents an elongated chain-like pattern which stems from the strong quasiperiodicity present in the corresponding time series. The five small modules in this network originate from the different amplitude levels generated by the Rossler attractor.

**Figure 6:**
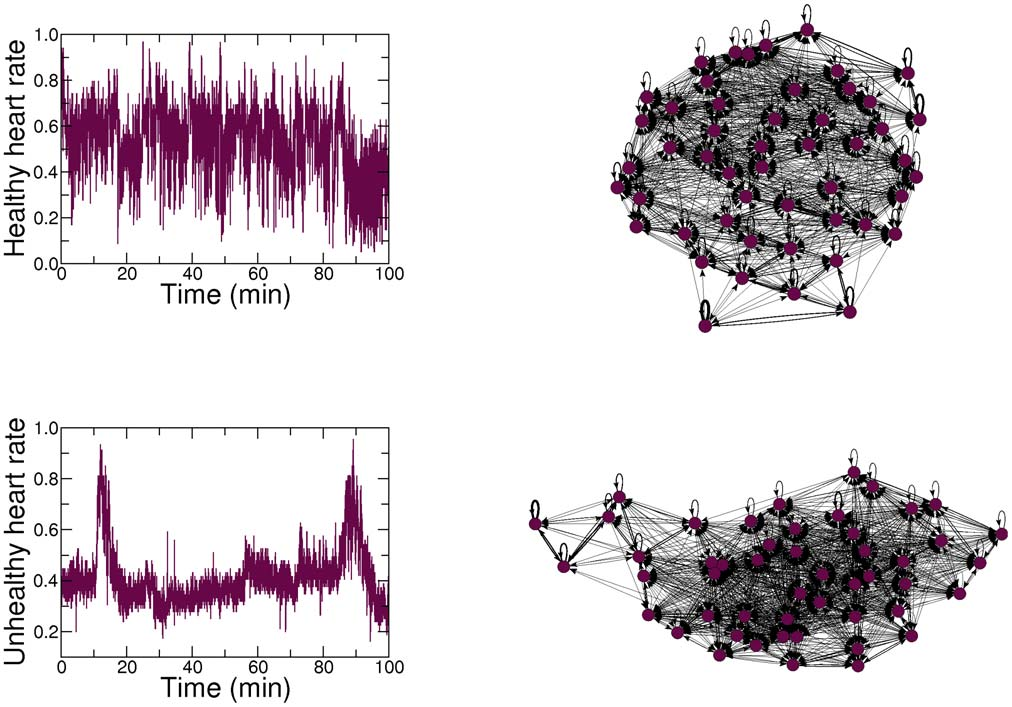
Illustration of the proposed forward map to the problem of detecting differences in the data structures of patients in different health conditions. We use 100-minute normalized heart rate time series from a healthy subject (upper panel) and a subject with severe congestive heart failure (lower panel) sampled every 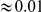 seconds (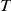 = 10,000 points) [36]. We construct the networks using 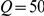 quantiles by applying 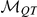 from the corresponding time series. The resulting networks display clear differences in topology, which are especially apparent on the relatively separated cluster in the network associated with the unhealthy subject. These differences in topology are confirmed by generating networks with different number of nodes (Fig. 7) and using time series from different healthy and unhealthy subjects (Fig. 8).

In order to further illustrate the potential for real-world applications of the forward map, we apply it to the long standing problem of detecting the subtle differences between interbeat interval time series of healthy and unhealthy subjects [35]. Specifically, we obtained two human heart rate time series from PhysioNet [36]; one from a healthy subject and one from a subject with severe congestive heart failure (Fig. 6). The healthy time series is notable for its apparent nonstationarity and “patchiness”. On the other hand, congestive heart failure may be associated with the emergence of excessive regularity, as is apparent from the unhealthy time series. We apply the forward map using 100-minute heart rate time series, 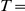
10,000 and 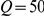 quantiles (Fig. 6). The resulting networks display clear differences in topology, which are especially apparent on the relatively separated cluster in the network associated with the unhealthy subject.

**Figure 7:**
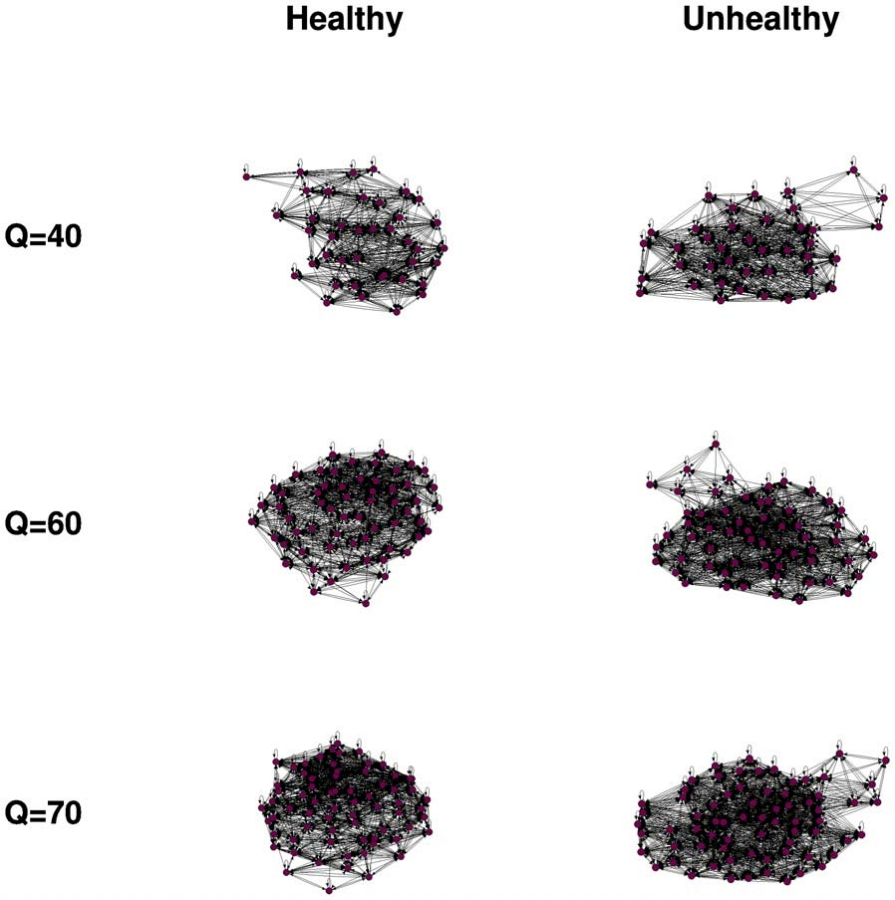
Application of the proposed forward map to the heart rate time series using different number of quantiles. We apply 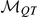 using 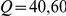 and 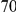 nodes to time series from healthy (left panels) and unhealthy subjects (right panels). Note the visual similarity of these networks with the networks presented in Figure 6, attesting the robustness of the results of the proposed forward map, regardless of the value of 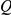.

**Figure 8:**
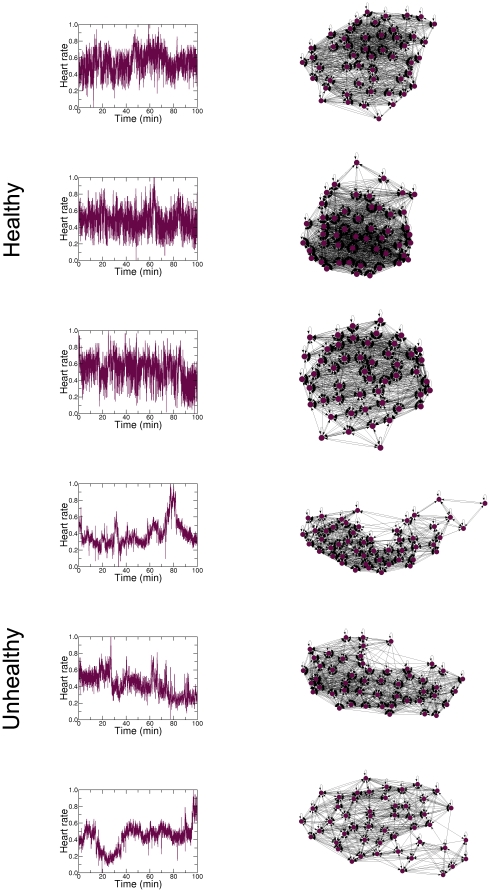
Application of the proposed forward map to the heart rate time series associated to different subjects. We apply 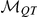 using 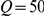 nodes to time series from three healthy (left panels) and unhealthy subjects (right panels). Regardless of the number of different subjects, the resulting networks are visually similar with those presented in Figure 6. This is another demonstration of the robustness of 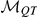.

We demonstrate the robustness of the results found in Figure 6 by applying 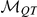 to the healthy and unhealthy heart rate time series over different values of 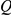. Figure 7 suggests that the forward map is able to produce networks with similar topologies, regardless of the value of 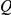. As another demonstration of robustness, we apply the forward map to the different healthy and unhealthy heart rate time series. Figure 8 suggests that the forward map is able to produce networks with similar dynamics for both healthy and unhealthy subjects.

**Figure 9:**
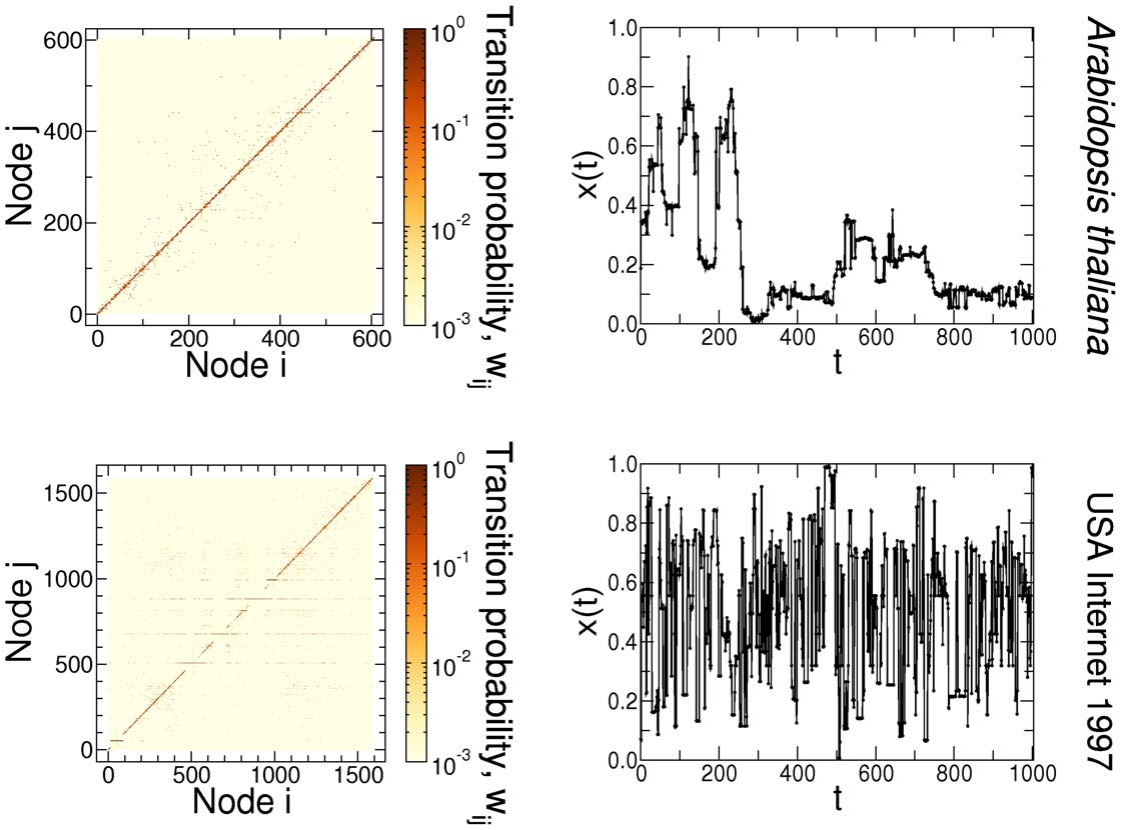
Illustration of the proposed inverse map to different types of real-world networks – metabolic network and the Internet. We use *Arabidopsis thaliana* network with 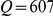 nodes and USA Internet 1997 with 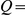
1,589 nodes [37], [38]. The corresponding adjacency matrices of these two networks are reordered (left panels) and times series with 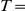
100,000 points each are generated by applying 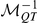 (for clarity only 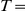
1,000 points are shown in the right panels). The resulting time series display clear differences in dynamics. In the first application, the topological features of the metabolic network are translated into a time-series with a high degree of persistence (or long-range correlations), due to the presence of modules in the original structure. In the second one, every time the random walker reaches one of the several hubs, it has a high probability of being sent to a different branch of the network. This behavior produces the noisy signal characteristic of low persistence (short correlations) time series. These differences in dynamics are confirmed by performing random walks over different realizations (Fig. 10), and computing their statistical properties (Fig. 11).

**Figure 10:**
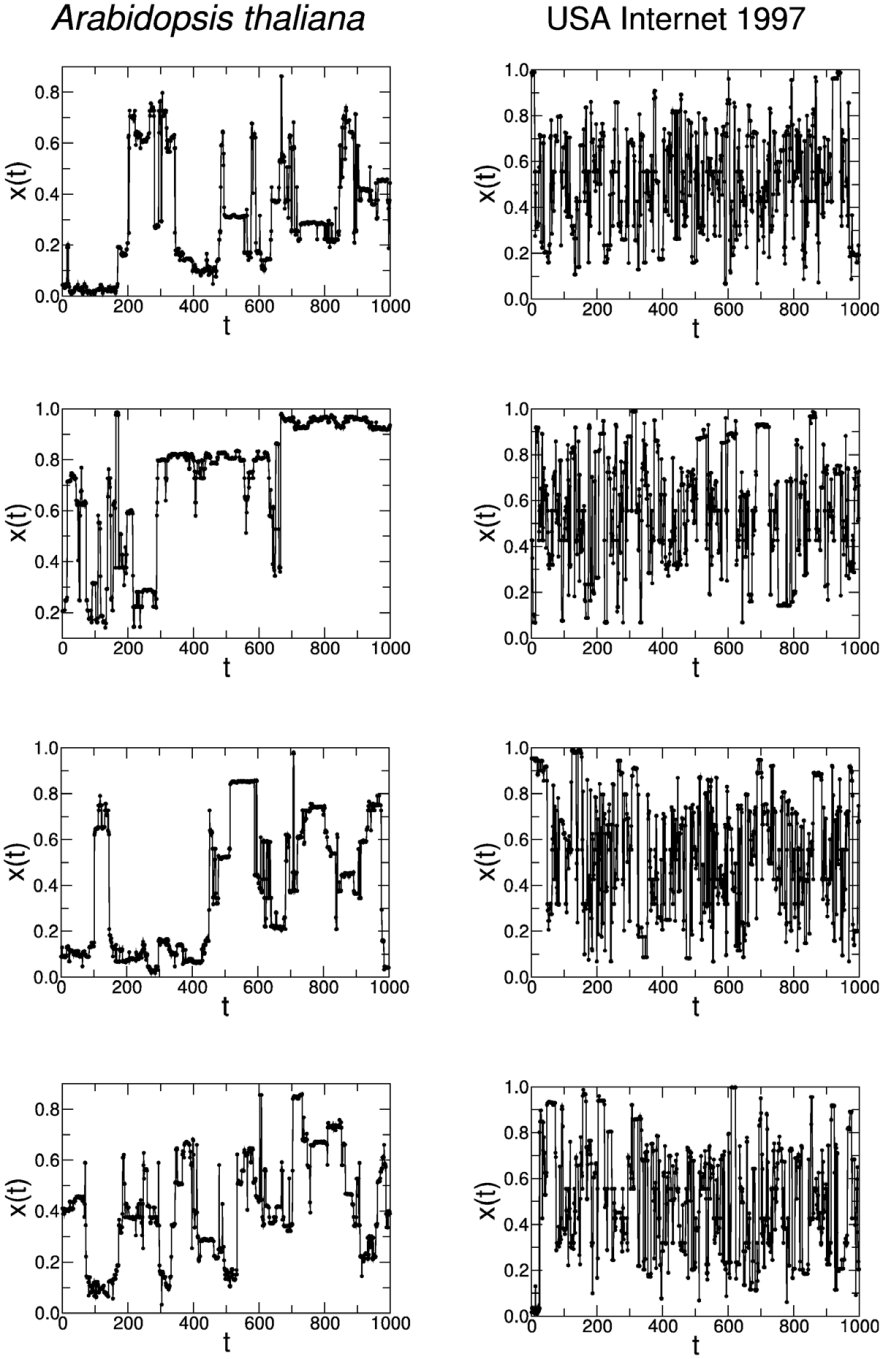
Different realizations of the inverse map 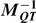 in the real newtorks. We perform four realizations of 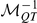 to the *Arabidopsis thaliana* metabolic network (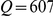 nodes and 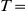
100,000 points), and USA Internet 1997 (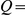
1,589 nodes and 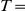
100,000 points). Note the clear similarity of these time series with the time series presented in Figure 9, demonstrating the robustness of the proposed inverse map.

**Figure 11:**
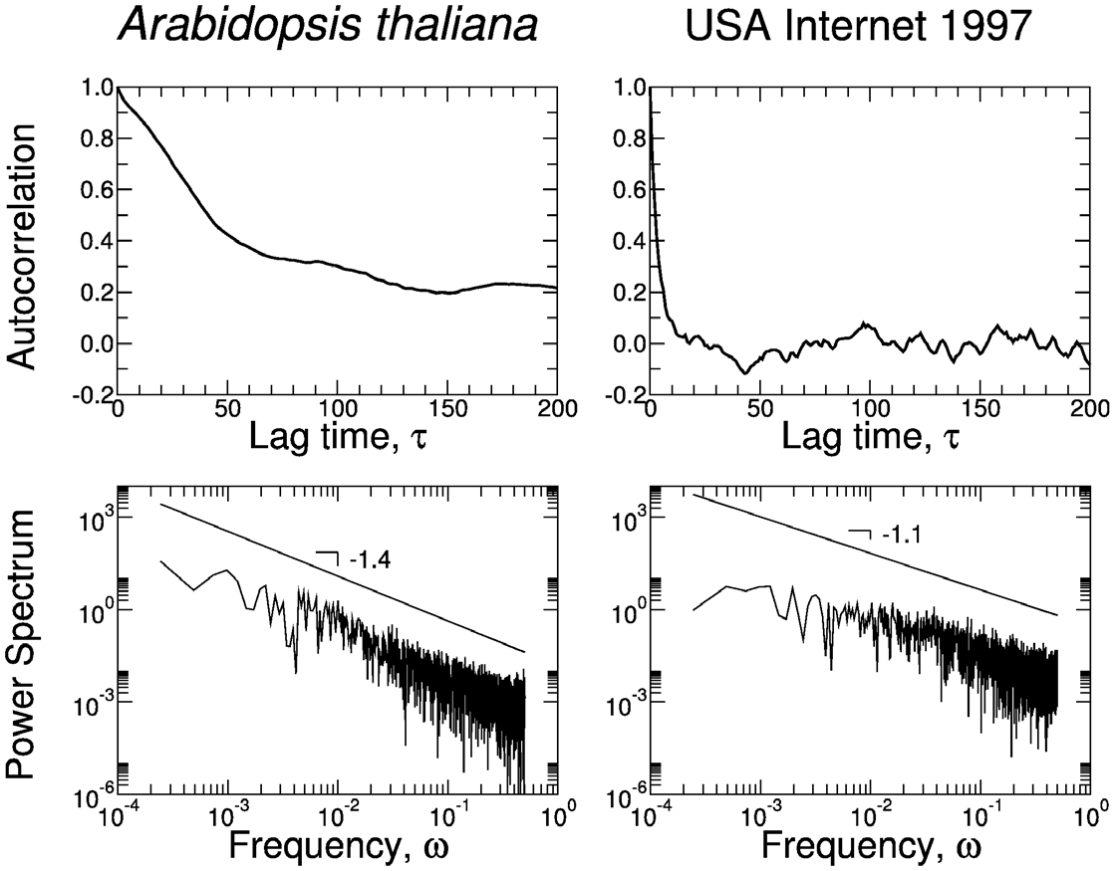
Statistical properties of the time series presented in **Figure 9**, generated from the *Arabidopsis thaliana* network and the USA Internet 1997. Note that the long-range correlations present in the metabolic network are well captured by the autocorrelation function and the corresponding power density spectrum, which displays a clear power-law scaling. On the other hand, the results in the USA Internet 1997 bear the footprint of the short-correlated signal generated by the Internet network. Note a power-law scaling with a less steep slope.

We also illustrate the potential for real-world applications of the inverse map described above by applying it to two networks belonging to different network classes (for details, see [37], [38]). The first network is the metabolic network of *Arabidopsis thaliana*, with a relatively high modularity, characterized by long open “chain” or closed loops of non-hubs, and a core of a few hubs that are directly reachable from one another. The second, the Internet in 1997, which has a star-like structure with several hubs and low modularity. First, we associate nodes to quantiles by reordering the corresponding adjacency matrices [29]. Next, we obtain time series with 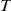 = 100,000 points each using networks with 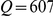 and 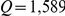 nodes, respectively (Fig. 9). The resulting time series display clear differences in dynamics, which we confirm by performing random walks over different realizations (Fig. 10), and computing their statistical properties (Fig. 11). Our results demonstrate that networks with different topologies result in time series with different dynamics.

## Discussion

The proposed map can be extended to include higher-order correlations. Just as a traditional Taylor expansion approximates the value of a time series 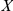 near a particular point 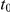 by evaluating the derivatives of 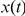 near 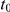, 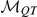 resembles a “wholistic” Taylor expansion – it estimates values near a particular point 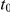 by the Markovian probability that 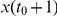 follows 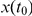 with the same accuracy for any point 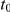 of the time series. Just as the precision of a Taylor expansion improves as higher-order terms in the expansion are retained, the precision of the map can be improved by incorporating higher-order Markov chains. For example, 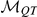 can be readily adapted to capture second-order correlations by constructing networks from the second order Markov probability density 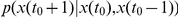, resulting in networks with directed and weighted hyperedges connecting the nodes associated with the quantiles of 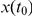 and 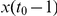 to the node associated with the quantile of 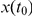.

It is worth mentioning that the proposed map procedure touches on a few classic analysis techniques. In some sense, it bears some resemblance to symbolic dynamics, where a continuous system is discretized into a sequence of symbols representing the state of the system [39]. In our map nodes play the role of symbols and a symbolic series is then produced by looking at a particular path through the network. The proposed map procedure also provides a unique approach to compressing time series data. Since most financial, health and climate time series consist of millions of measurements, our map procedure naturally provides an excellent storage mechanism to compress the 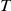 points of these large time series into a list of at most 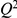 values of the Markov transition matrix 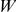. Additional storage savings occurs when 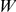 is sufficiently sparse that it is more efficient to store a weighted edge list.

Our results build a bridge connecting time series analysis and network-related research. In this sense, networks can be analyzed by exploring an extensive set of statistical properties of the associated time series. For example, motifs in a network are mapped as periodicities in a time series, which are characterized by looking at the corresponding power spectrum of the time series. At the same time, different dynamical regimes in time series can be analyzed by exploring an extensive set of topological statistics at the associated network domain.
